# Modified Provisional Technique: A New Prosthetic Approach for Stable Gingival Displacement to Make Impression

**DOI:** 10.1155/ijod/2998042

**Published:** 2026-02-27

**Authors:** Maurizio De Stefano, Mario Papa, Antonio Rupe, Alfonso Acerra, Francesco Giordano

**Affiliations:** ^1^ Department of Medicine, Surgery and Dentistry, University of Salerno, 84084, Salerno, Italy, unisa.it

**Keywords:** digital impression, gingival displacement, prosthetic technique

## Abstract

Making impressions of abutments prepared using the subgingival vertical preparation technique in full‐mouth rehabilitation cases is challenging, both in terms of bleeding control and effectiveness of gingival displacement. Although gingival retraction techniques include the use of gingival cords and astringents, which represent the current gold standard, they do not guarantee the opening of the gingival sulcus to ensure an uninterrupted reading stream of all the arch elements. In fact, once the retraction cord is removed, the gingival sulcus tends to revert to its original position after the initial 60 s, thereby preventing the detection of the subgingival preparation of most elements in the full arch by the scanner. Thus, to ensure precise recording of the subgingival preparation of all the arch elements, various scanning software techniques, such as cutting and stitching the areas of the distorted impression, have been devised. The prosthetic technique described in this article uses modified provisional restoration in its margins, thereby allowing impression making of individual teeth or the full arch. Upon removal of the provisional restoration, gingival displacement exposes the subpreparation zone, with pink and nonbleeding gingiva, which maintains its stability beyond 5 min. This technique allows immediate recording of the full arch, thereby reducing the scan time in the absence of the use of cords and anesthesia, interference at the apical one‐third, interruptions, mesh hole creation, and overlapping seam. Thus, this technique improves overall patient compliance and the chairside time.

## 1. Introduction

Impression making is a process needed in many different cases in dentistry. The aim of an impression is to transfer the information from the patient to the clinician. Nowadays, the gold standard is the conventional impression that can be made with different impression materials and using different trays. Subsequently, the impression will result in a gypsum cast [1]. During the years, a different method to make impressions has been developed, that is, the 3D intraoral scanner (IOS) without impression materials [2]. These scanners are used to obtain digital dental models directly from the patient for different purposes [3, 4]. This capture can be achieved through different methods, such as direct scanning of the oral cavity, impression scanning, which consists of indirect acquisition of the arches through impressions made in the traditional way, and finally model scanning; and in this case, the scanning is done on the model after it has been developed from impressions acquired in the traditional way [5]. The advantage of the latter method is that the technician, having a real model, can work in advance of the scan to rectify inaccuracies. Whatever the type of scan performed, the result is the same, a virtual model, that is, a drawing in three dimensions that reproduces what was scanned. There are several traditional impression techniques to make a single tooth impression. In general, the most used technique is the monophase and extra light body in a sectional stock tray. A data analysis has been made to evaluate that a different impression technique using a matrix is the best way in terms of accuracy and dimensional stability [6]. There are different materials to take dental impressions, such as alginate, that is used for less accurate impressions such as bites for bruxism or in case of whitening processes, putty and light body materials that have different chemical compositions and are used for more precise impressions and are more stable over time compared to alginate, which needs to be quickly transformed into a gypsum cast in order to keep its volume. The combination of putty and light body can give different details to the impression [7]. In the case of a full‐arch rehabilitation, there are two different methods to take an accurate impression: the conventional one (using impression materials and customized trays) or the 3D IOS technique. A randomized clinical trial by Cappare et al. [8] states that the IOS can be a reliable alternative for implant full‐arch rehabilitation thanks to its precision. Impression making of the dental arches in full‐arch rehabilitation cases is a complex step requiring clinical expertise [1]. The accuracy of the impression depends on the impression technique and the management of gingival displacement, mouth opening, bleeding control, and sulcus width over time, especially when several dental elements need to be recorded [9].

Dental impressions are the negative imprint of hard and soft tissues of one or both arches, and they allow a plaster model to be formed, that is, a positive reproduction. Traditional dental impressions can be made of different materials, such as alginate, while digital impressions are captured by an IOS [10].

The shift from conventional to digital impression‐making techniques had many advantages, including a reduced chairside time and improved patient compliance [11]. On the other hand, the use of IOSs in clinical settings can be complicated by several factors, such as the humidity in the oral environment, presence of saliva and bleeding, patient movement, and scanning technique [12].

Direct light access over the object to be scanned is necessary for the precise recording of narrower areas, such as those within the gingival sulcus [13]. Even after optimal control of these factors during impression making, the depth of the finish line within the sulcus may adversely affect the quality and accuracy of the digital impression [14]. Light entry can be influenced by the distance between the scanner and the subgingival preparation, proximity of the soft tissue to the abutment, and the angle of light incidence. However, the unfavorable orientation of the handpiece results in image distortions that significantly compromise the accuracy of the impression. In addition, IOSs are technique sensitive [15].

Although IOSs can be favorably employed for making impressions of single dental elements for fabricating interim and final prostheses, less satisfactory results have been demonstrated in cases of full arches or the presence of several dental elements when compared to those using conventional impression techniques [16]. Computer‐aided design (CAD) and computer‐aided manufacturing (CAM) laboratory studies have demonstrated that impressions made using IOS systems on multiple tooth elements result in less accurate outcomes, such as models and dental crowns, compared to those obtained from conventional impression‐making techniques [16, 17]. In full‐arch rehabilitation cases using the subgingival vertical preparation technique, making the final impression immediately after abutment preparation is unlikely because of the challenges associated with the management of certain factors, such as bleeding, that adversely affect the quality of the impression. Thus, tissue healing should be managed by incorporating provisional restorations and using a postprovisional impression technique. Gingival retraction, which often requires anesthesia, constitutes an important step at this stage of the prosthetic workflow. Currently, the double‐cord technique with the use of astringent liquid is considered the gold standard when compared to other techniques involving the use of retraction pastes or surgical techniques [18]. Although the use of pastes can render gingival retraction less traumatic, it is less effective in dilating the sulcus and ensuring recording of the subgingival margins of the prepared tooth within the sulcus. Gingival retraction should be effective in terms of both dilation and adequate duration for recording a full arch [19]. The double‐cord technique allows a sulcus opening of 1 mm compared to that of 0.4–0.7 mm by pastes. Although both techniques have demonstrated effectiveness in maintaining the sulcus opening in the first 40 s for making the impression, only the cord technique can maintain an adequate sulcus opening up to 60 s [20, 21]. Once this time limit is exceeded, rapid closure of the space obtained by gingival retraction occurs, compromising the subgingival margin detection. Although the closure of the sulcus slows down, the initial dilatation is lost, which can result in grave inaccuracies in the final impression, especially when recording several dental elements. Compared to the conventional impression‐making technique, the use of digital impressions can solve these problems by cropping certain areas of the scan, which can subsequently be rescanned individually, reading each tooth after removing the retraction cords, thereby maintaining gingival displacement [22]. Nevertheless, impressions with one or more mesh holes are less accurate than those made using a single, uninterrupted reading stream. This lower accuracy could be attributed to various scans undergoing stitching procedures by the software [23–25].

Keeling et al. [14] demonstrated that supra‐gingival or juxta‐gingival preparations are easier to read when combined with digital scanning techniques than those with subgingival margins. Indeed, many studies advise against the use of digital impressions for recording tooth preparation with a subgingival finish line with a depth of 1.5–2 mm [26, 27]. Moreover, Keeling et al. [14] suggested that the distortion of digital impressions is due to the absence of direct light access in certain areas, so that the low density of information received requires software adjustment of the final curvature value, thus resulting in a less accurate impression [28, 29].

Thus, the accuracy of digital impressions of subgingival preparations in full‐arch cases is uncertain. Some authors have proposed a technique without incorporating the commonly used gingival retraction techniques and instead using the emerging profile of the provisional restoration itself as a spacer [30, 31].

The purpose of this article was to describe a clinical procedure workflow that allows the complete dislocation of the free gingival margin to better detect the limit of the subgingival vertical finish line, to obtain an accurate digital impression, without the use of retraction techniques. This technique is particularly proposed when numerous abutment teeth are subject to the impression making.

## 2. Technique

1. Perform a circumferential probing to map the sulcus of the teeth to be prepared.
2. Perform a subgingival vertical preparation (Figure 1A). Insert a #000 retraction cord (Ultrapack, Ultradent) soaked in an astringent if the aim is to prepare with a finish line. Do not insert any cord if the aim is to prepare a vertical edgeless preparation.
3. After the tooth preparation, in case of preparation with a finish line, insert a second retraction cord #0 or #1. In case of preparation without a finish line, insert a first retraction cord #000 soaked in astringent and then a second retraction cord #0 or #1. This ensures adequate intrasulcular space that can be completely penetrated by the resinous materials used for the relining of the provisional restoration, so as to dislocate the gingival margin of the sulcus completely (Figure 1B).
4. Fill a disposable syringe with mixed resin (Sintodent s.r.l.) and inject it into the provisional restoration, made of polymethyl methacrylate resin. Remove the second retraction cord or both cords if the sulcus is shallow, then isolate the abutments with liquid glycerin. Fill the sulcus completely with the flowable acrylic resin, so that the area beyond the apical limit of the preparation, more easily defined as the “subpreparation zone” is also read, and reposition the provisional restoration on the abutment teeth (Figure 1C). At the end of the procedure, two circumferential areas can be observed. The first is the internal circumferential area, indicating the end of the sulcus, while the second is the external circumferential area, indicating the gingival margin. The two lines thus delineate a circumferential concavity (Figure 1D).
5. Remark the inner edge with a pencil and fill the circumferential concavity completely with flowable acrylic resin (Figure 2A–C).
6. Refine the provisional restoration with the aim to obtain a 90° angle margin: the average thickness of the vestibular/palatal area should be 1 ± 0.5 mm, while the thickness of the surrounding areas should be between 0.5 and 1 mm, based on the position of the tooth in the arch and the actual approximate space available (Figure 2D).
7. Try the provisional restoration, polish it, and cement it with temporary cement (Temp Bond NE, Kerr). After 2 min, remove the provisional restoration and then the excess cement, beyond the axial walls, so that the clean provisional restoration is in contact with the gingival tissue (Figure 3A). Then place the provisional restoration using only the Temp Bond cement activator (Figure 3B).
8. After 1 week, remove the provisional restoration, remove the cement residues from the abutments with a silicone tip (Opti Clean, Ivoclar), rinse and dry the abutments, and make the final impression with an IOS (TRIOS 3; 3Shape) (Figure 3C,D). In the same session, modify the provisional by conforming a conventional emergency profile, eliminating the 90° margin.

Figure 1(A) Upper arch teeth treated with vertical subgingival preparation. The gingival tissues are not in favorable conditions to make the final impression. (B) Insertion of the retraction cords before relining the provisional restoration in order to dilate the sulcus. This process allows the fluid resin to read the entire sulcus. (C) Provisional restoration relined with a positional guide. (D) Two circumferential areas can be observed. The first is the internal circumferential area, indicating the end of the sulcus, while the second is the external circumferential area, indicating the gingival margin. The two lines thus delineate a circumferential concavity.

**Figure:**
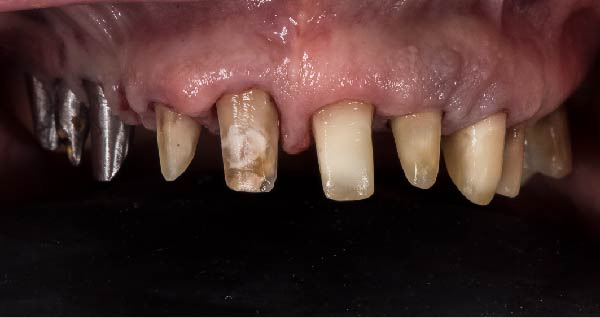
(A)

**Figure:**
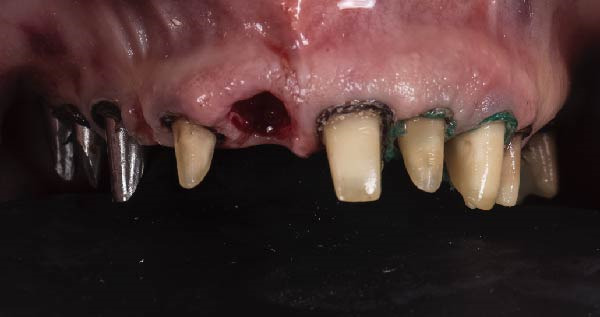
(B)

**Figure:**
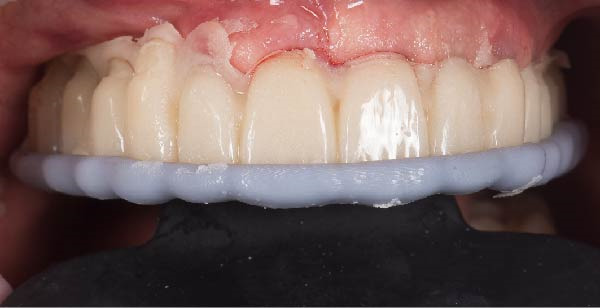
(C)

**Figure:**
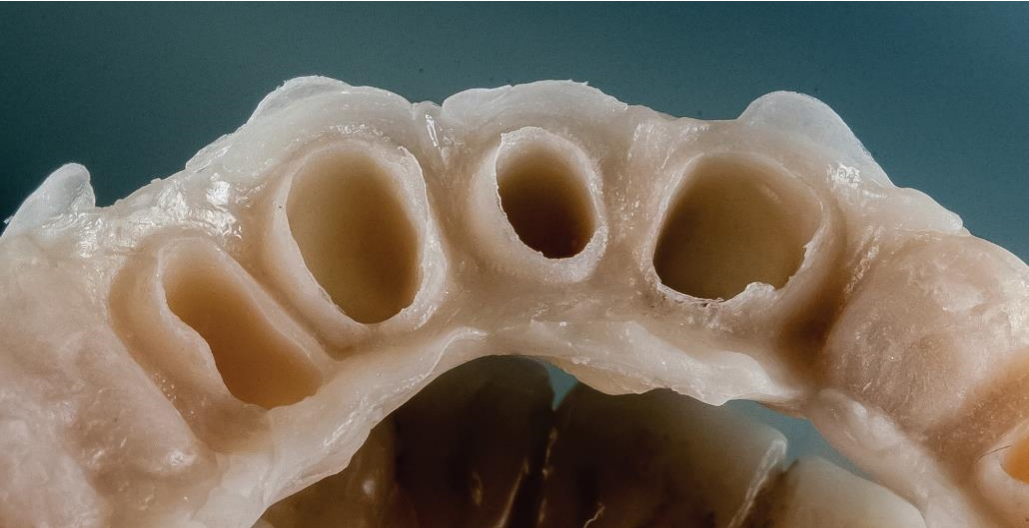
(D)

Figure 2(A) The bottom of the sulcus read by the fluid resin can be highlighted with a pencil. (B) The concavity between the two circumferential areas is filled with fluid resin. (C) The recontouring is completed with the aim to fill the entire circumferential concavity. (D) Provisional restoration refined according to the new technique, with a 90° angle and 0.5–1.5 mm thickness.

**Figure:**
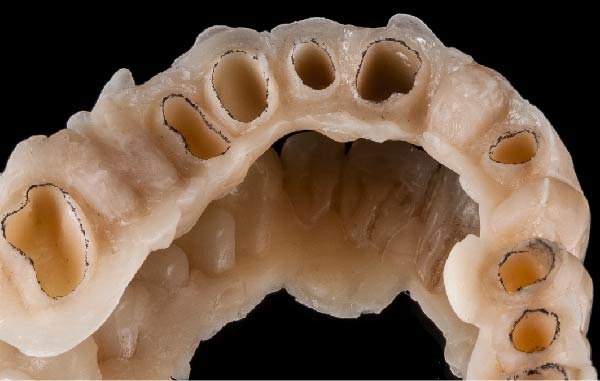
(A)

**Figure:**
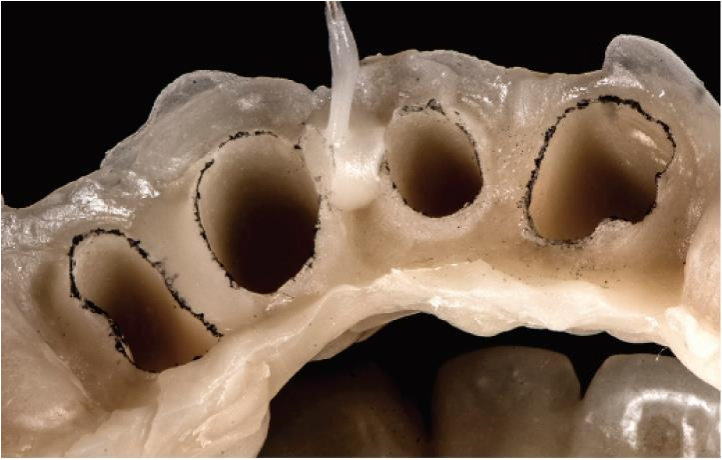
(B)

**Figure:**
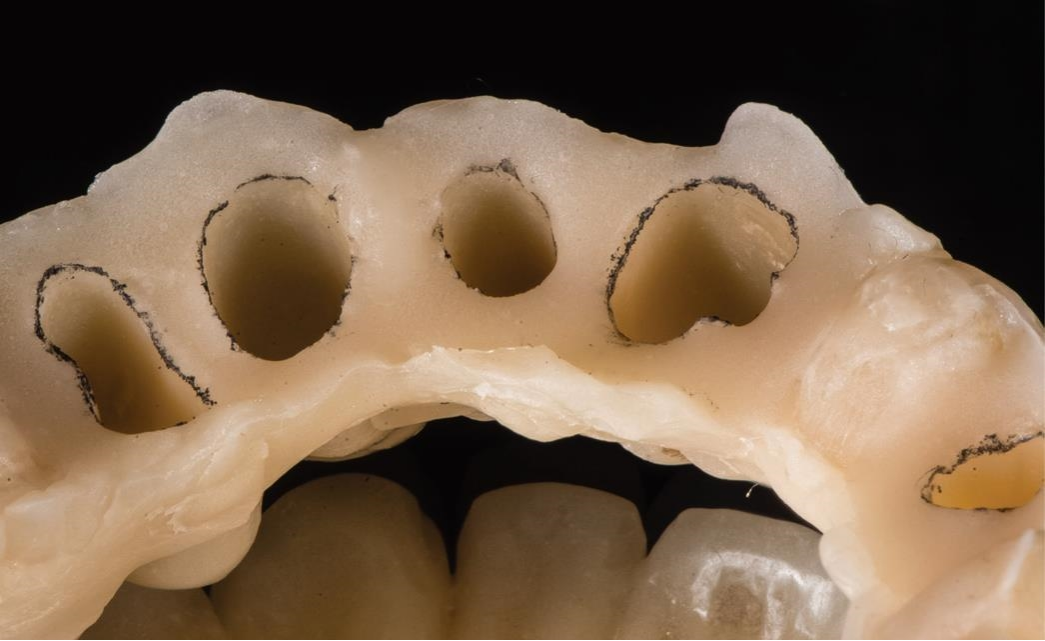
(C)

**Figure:**
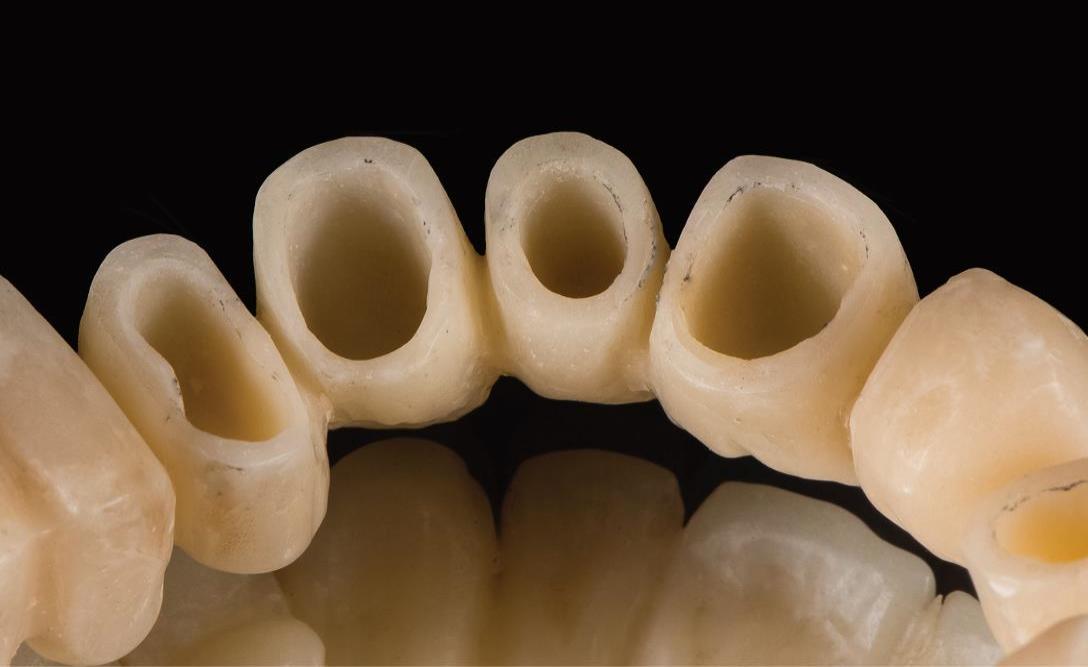
(D)

Figure 3(A) In this technique, the operator removed the interim crown outside the mouth after cementation to clean the excess cement. This step allows remove of all the cement in every area, enabling optimal healing of gingiva, long‐term stability of the gingival tissues, and preventing localized bleeding. (B) The provisional restoration is repositioned using only the cement activator. (C) The aspect of the gingival tissue 7 days after the relining prosthetic procedure. The provisional is detached, the abutment teeth are cleaned, and the final impression is immediately made with an intraoral scanner (TRIOS 3, 3 Shape). (D) This technique, called by the authors the “Sulcus zeroing technique” (SZT), allows the exposure of the cervical limit of the preparation and to make an impression without interference to the light of the digital scanner.

**Figure:**
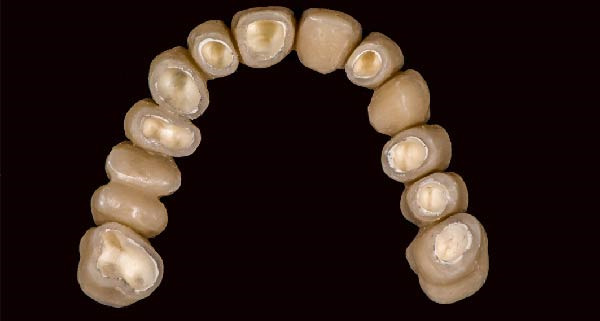
(A)

**Figure:**
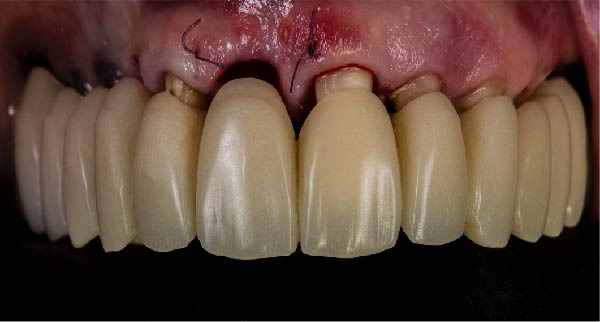
(B)

**Figure:**
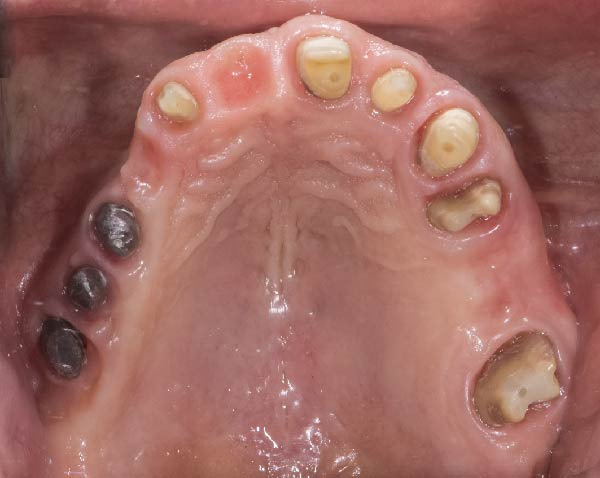
(C)

**Figure:**
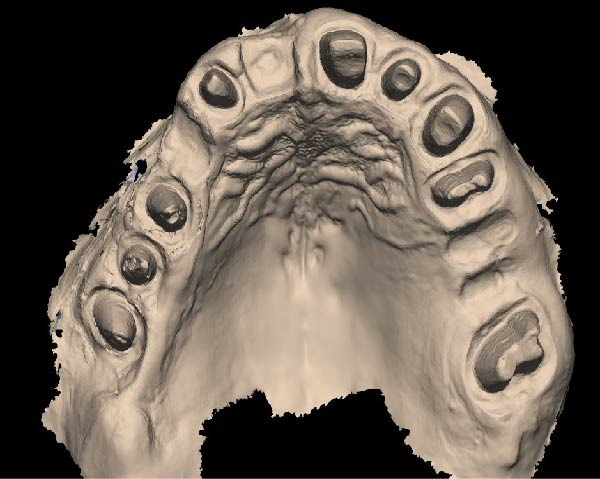
(D)

## 3. Discussion

The technique described in this paper allows making impressions of single teeth or full arches using a postprovisional technique by detaching the provisional restoration and directly reading the full arch in a short time without using cords or anesthesia, and in the absence of interference at the apical one‐third, interruptions of the handpiece, mesh hole creation, and overlapping seams. Upon removal of the provisional restoration, gingival displacement completely exposes the preparation finish line and the subpreparation zone in case of vertical subgingival preparation with a finish line. The same technique provides reading of the full vertical area of prosthetic closure in case of vertical preparation without a finish line, allowing it to be easily read using the IOS. The gingival tissue appears stably flattened, eutrophic, and free of inflammation, corresponding to a geometry mirroring the 90° contour accomplished by the relining. The latter is crucial for vertical compression of the gingiva, rather than lateral tension of the connective fibers. The thickness of the provisional restoration margin was managed differently depending on its anatomical position in order to respect the biological width. In particular, where the thickness of the provisional restoration margin is greater than the horizontal width of the gingiva, such as the buccal and linguo–palatal area of thin biotypes in the lower incisal group, the provisional restoration shows a flat profile. In areas where the provisional restoration margin is thinner than the gingiva, mainly in approximal areas and thick biotypes, the gingiva shows a shape that mirrors the 90° profile of the provisional restoration (Figure 4A,B). A minimum margin thickness of 0.5 mm was observed because this was the lowest thickness that could give an effective and stable gingival displacement and could consent to light penetration of the IOS scanner.

Figure 4(A) The gingival tissue aspect obtained with the new technique is schematized: when the gingiva is thick, the contour of the provisional restoration leaves a 90° shape. (B) When the gingiva is thin, as with the lower incisors, the provisional recontouring allows the gingiva to show a shape that mirrors a flat profile of the provisional restoration.

**Figure:**
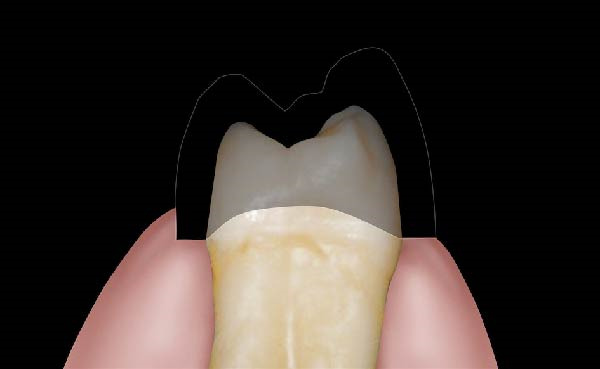
(A)

**Figure:**
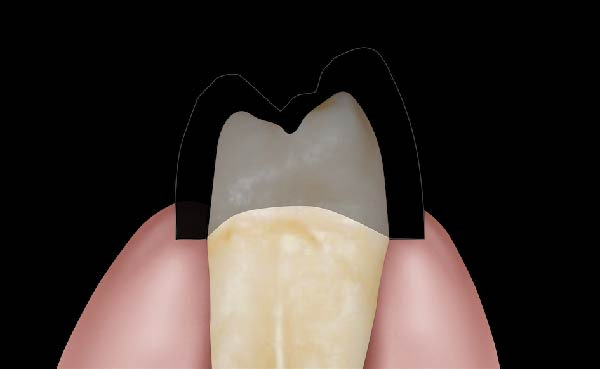
(B)

Upon detachment of the provisional restoration, removal of the temporary cement and rinsing of the abutments were not associated with gingival bleeding. The manual act of impression making is simplified, as the sulcus is stable and open for the duration of impression making, even well beyond the clinical time required to make full‐arch impressions. The position of the scanner during impression making is orthogonal to the abutment, even at the most apical area of the preparation (Figure 5A,C). This applies both in cases of vertical preparation with a finish line, where the clinician needs to read the subpreparation zone, and in cases of vertical preparation without a finish line, where the clinician needs to read the full available vertical area of prosthetic closure.

Figure 5(A and B) Buccal and occlusal view of a tooth treated with the new prosthetic approach. The gingival shape and its stability can be observed. When the provisional is detached, the sulcus is immediately ready to be scanned without any retraction technique. (C and D) The three‐dimensional model shows how the dental technician can mark the finish line of the preparation without the interference of the gingival tissue.

**Figure:**
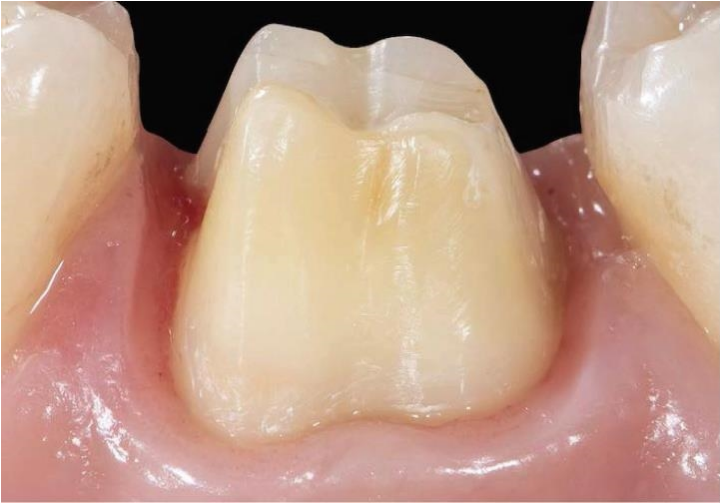
(A)

**Figure:**
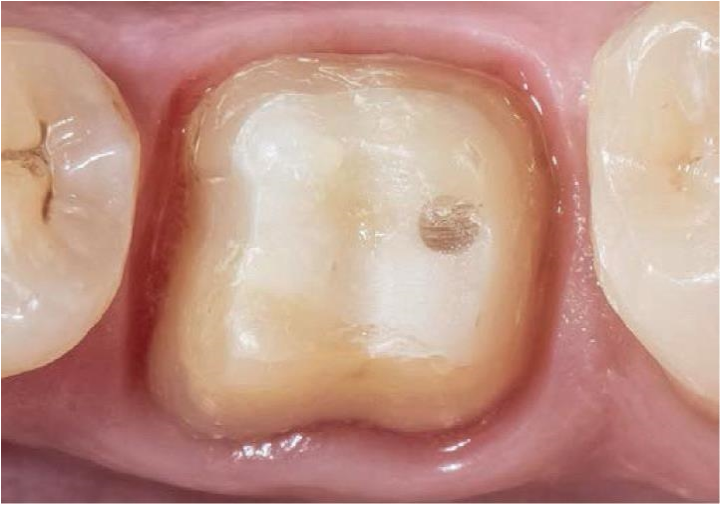
(B)

**Figure:**
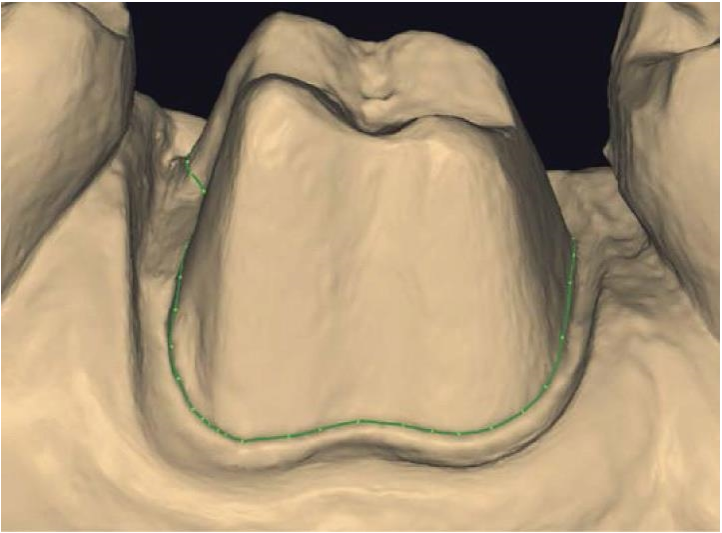
(C)

**Figure:**
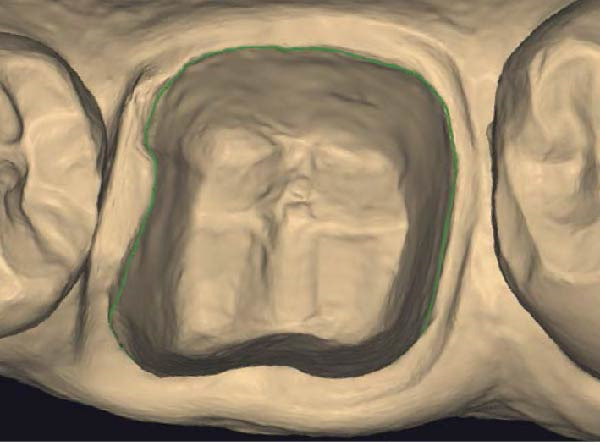
(D)

Reading is performed without interrupting the scan flow, as the sulcus remains open and stable throughout the scanning of all elements, without the need for creating mesh holes and stitching of new scans in the point cloud of the original file (Figure 5B,D). The prosthetic technique has certain significant clinical advantages over other methods currently employed in making full‐arch digital impressions. The clinical phase of the impression does not require the use of retraction cords or astringent pastes; consequently, anesthesia is not required during the procedure.

The maximum time for making impressions of full arches, after detaching the provisional restoration, was 3 min and 30 s, demonstrating a significant reduction in the average chairside time. Patients reported no discomfort, only slight thermal sensitivity to rinsing and cement removal from vital abutments, thus showing good compliance.

The techniques currently described in the literature, such as Di Fiore et al. and Schmitz et al., where the provisional restoration is used as a spacer in a vertical subgingival preparation to avoid the use of retraction cords, do not always render the possibility of reading the subpreparation zone [30, 31]. Moreover, in any case of very vertical intrasulcular preparations, which are always accompanied by a finish line, detachment of the provisional restoration and immediate impression making does not guarantee precise reading of the emergence profile of the tooth by the dental technician. Thus, confirming the precise fit of the restoration margins to the finish line may not be possible. However, the technique described in this article, which ensures gingival displacement to its full depth, allows the subpreparation zone to be read even in cases of very deep subgingival preparations of 1.5–2 mm. Thus, an impression with a clear readout of the finish line is provided to enable precise ditching on the abutment model and accurate final emergence profile.

**Figure 6:**
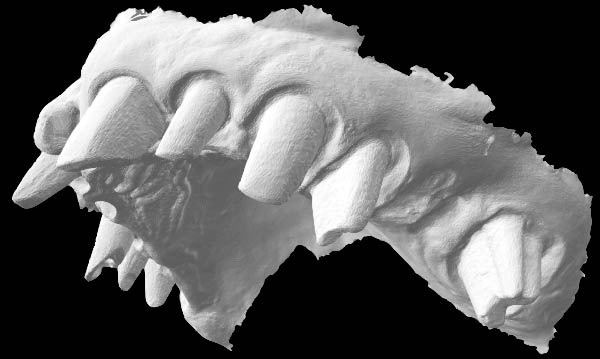
A standard tessellation language file (STL). The absence of the gingiva near the abutment teeth is a factor that allows the clinician to make the impression without interruption of the scanner flow.

Compared with other impression‐making techniques, in the present prosthetic technique, the vertical and horizontal stress directed toward the free gingiva, result in gingival tissue maturation that adapts to the 90° margin of the interim crowns. This way, when the interim crowns are removed, the gingiva is already completely displaced, without any retraction technique. This allows the light beam of the IOS to enter freely and orthogonally and to have the mesh with a correct curvature value, even on the most delicate areas of the subgingival preparation (Figure 6).

The gingival tissue upon detachment of the provisional restoration looks very similar to the tissue observed upon unscrewing the healing abutment from the implant following completion of healing. The gingival tissue is stable throughout the duration of the impression making, unlike the limited time available with conventional gingival retraction techniques.

Only 60 s of stable gingival retraction is possible using the double‐cord technique; thus, continuously scanning a complete arch in a single scan is impossible. Moreover, once the retraction cords are removed, the gingiva undergoes premature sulcus closure in most dental elements before the scanner light can read the final preparation of all the elements.

Thus, some authors have taken advantage of the ability of IOS software to create mesh holes in the preexisting scan and then rescan the anatomical parts that are not congruent, rescanning the mesh holes. However, cutting and rescanning mesh holes have been shown to negatively affect the accuracy of IOS scanning, as demonstrated by Gòmez‐Polo et al. [23] in their study.

**Figure 7:**
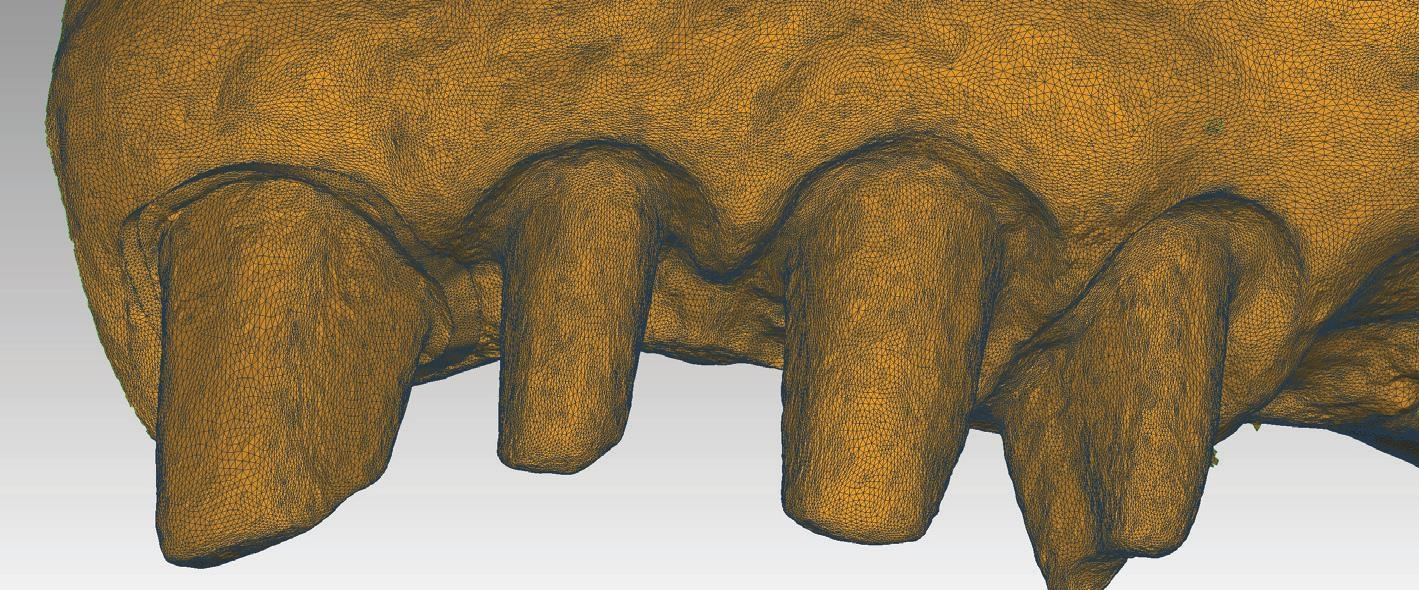
The displacement of the sulcus, even beyond the finish line of the preparation, allows the clinician to utilize the scanner handpiece in the best possible orthogonal position for all abutments. Consequently, the point cloud obtained in the STL file does not show any loss of density, even in the cervical part of the dental preparation.

Then, Valenti et al. [22] have considered a reverse scanning technique for making impressions of full arches, beginning with the provisional restoration and then using the software’s tool to create holes in the mesh on all the abutments. Each abutment is then individually rescanned and locked [22]. In contrast, the technique described in this article is less time‐consuming and does not require management of single‐tooth gingival displacement, either clinically or on the IOS software. The prosthetic technique described in this paper does not involve these steps. In addition, stable gingival displacement of more than 5 min has been observed using this technique, bypassing the limits given by other techniques using retracting cords. This time was measured by serial scans of the abutments performed every minute from the first to the fifth and overlaying these scans to verify the stability of gingival displacement. Thus, smoother scanning with no stitching‐related overlaps is possible, thereby making this new technique subject to fewer variables (Figure 7). The entire impression‐making procedure also reduces the clinician’s learning curve in presumably the most delicate phase of prosthetic rehabilitation.

Although the technique described was designed for cases of vertical tooth preparation, this new approach of modified provisional concepts could be used in cases of horizontal preparation. Further researches on this technique are warranted to observe the actual duration of gingival displacement following detachment of the provisional restoration, which currently already exceeds the time required for impression making of full arches. Further studies are certainly needed to assess the risk of tissue trauma from repeated removal of the provisional and to document the actual timing of gingival rebound subsequent to cementation of the interim restoration, after elimination of the 90° margin, in favor of a conventional edge. However, current clinical observations at 72 h after delivery of the interim prosthesis show stable tissue recovery, including the presence of the new gingival margin position and gingival papillae (Figure 8A–D). The gingival tissue readjusts to the new emergence profile of the crown, and no incidence of gingival recession has been noted in any case. Despite clinical observations, which also demonstrate tissue stability (Figure 9), statistically significant samples are being investigated in other studies to validate the absence of complications.

Figure 8(A) Digital model obtained after the impression was made with the dislocation of the provisional restoration with the 90° angle (brown model). Immediately after detecting the digital impression, the 90° angle of the margin of the provisional restoration is modified by creating an edge with a conventional emergency curvature. With this step, gingival tissue maturation moves in centripetal–coronal direction, and it adapts to the new forms of the interim crowns. After 72 h from the modification, in order to leave space and time for the tissues to return, it is possible to make a new digital impression of the refurbished tissues. (B) Digital model of gingival tissues that have adapted to the new emergency profile of the interim crowns, recreating, thanks to the coronal movement of the gingiva, new free gingival margin profiles and papillae (pink model). (C) Overlapping of the two models (A and B). The gingival tissues returned to position after the modification on the provisional restoration (pink model) are in excess and detached from the underlying brown model, where the gingival sulcus was dislocated completely. The limits of the two gingival margins of the overlapping digital models will serve as an indication to decide where to place the finishing line of the final restoration. (D) A section of the overlapping of the two models (A and B). It is evident, in the design of the pink section model, that the tissue has moved almost 1 mm in the coronal direction. In addition, the gingival thickness in the vestibular part has reduced in bucco‐palatal dimension (yellow section) in favor of increased coronal height (pink section).

**Figure:**
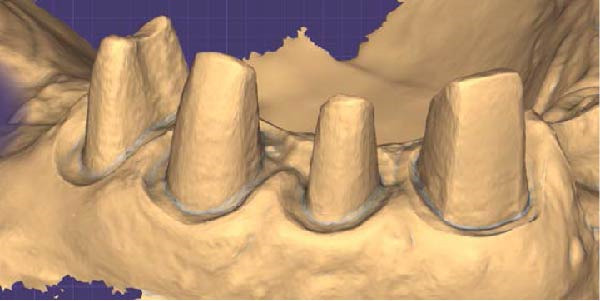
(A)

**Figure:**
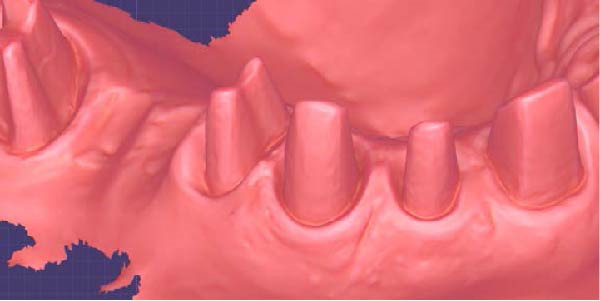
(B)

**Figure:**
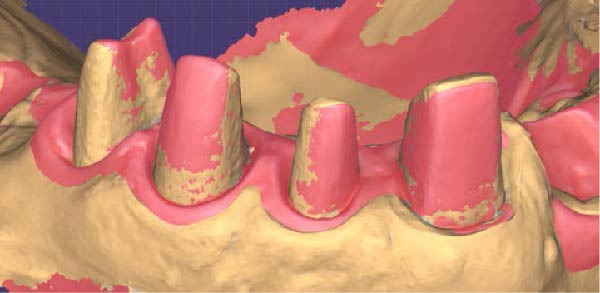
(C)

**Figure:**
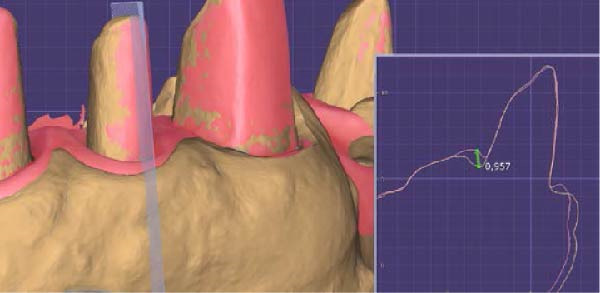
(D)

Figure 9(A) Final prosthetic rehabilitation just cemented. (B) Clinical 2‐year follow‐up.

**Figure:**
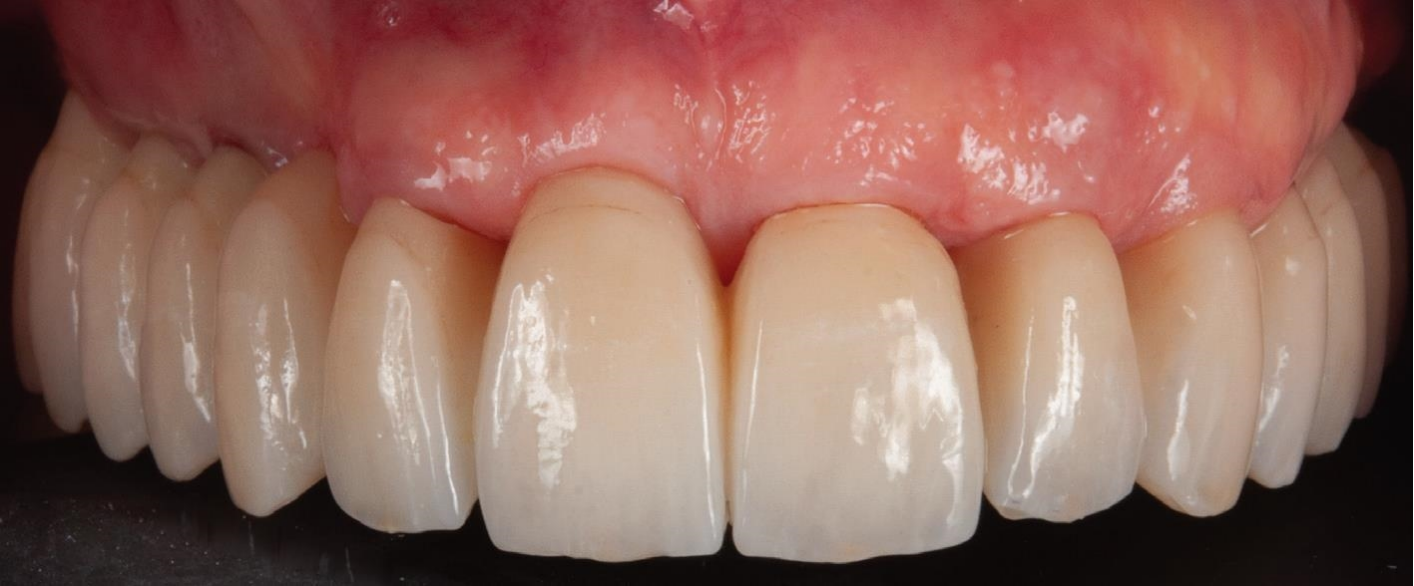
(A)

**Figure:**
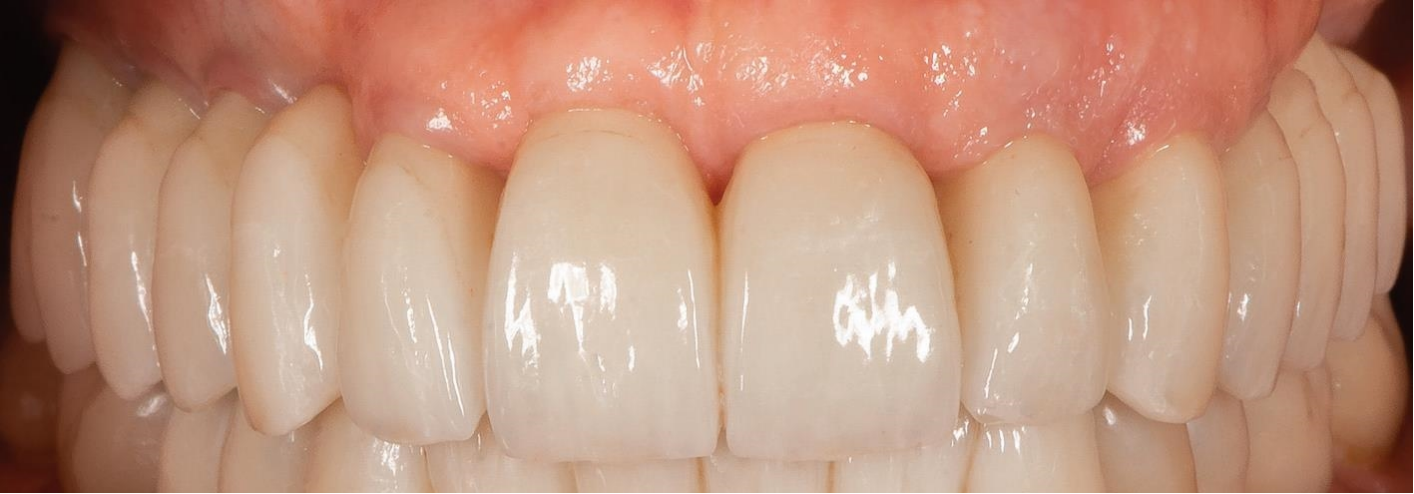
(B)

## 4. Conclusions

This new technique of modifying the provisional restoration margin allows the clinician to detach the provisional restoration and directly make digital impressions of single dental elements or full arches using a post‐provisional technique. It is especially indicated in cases of full‐arch rehabilitations, where gingival retraction for a longer period of time is required to manage the factors, such as gingival bleeding, currently complicating the clinical procedure and accuracy of the final impression. It does not require anesthesia or the need for insertion of retraction cords with astringent to take the impression, thereby improving patient compliance; it also seems to be indicated in cases of deep vertical preparations over 1.5–2 mm. It appears to reset the sulcus to zero, allowing the free gingiva to be expanded and stabilized for over 5 min, thereby allowing positioning of the scanner light orthogonal to the cervical axial wall on all the dental elements to be scanned; mesh holes would not need to be created in the point cloud, thereby eliminating errors related to rescanning and stitching, and consequently greatly reducing the chairside time; in cases of vertical preparations with a finish line, the subpreparation zone can be detected by gingival displacement of the entire sulcus, thereby indicating the correct emergence profile and marginal closure line of the final restoration.

## Author Contributions

**Maurizio De Stefano:** conceptualization, methodology, investigation, data curation. **Mario Papa**: software resources, data curation, writing – original draft preparation. **Antonio Rupe**: software investigation. **Alfonso Acerra**: validation, writing – review and editing, visualization, supervision. **Francesco Giordano**: validation, visualization.

## Funding

This research received no external funding.

## Ethics Statement

The authors have nothing to report.

## Consent

The authors have nothing to report.

## Conflicts of Interest

The authors declare no conflicts of interest.

## Data Availability

No data are in this technique article.
